# Preliminary Study on the Use of *Sargassum horneri* as a Dietary Ingredient for Juvenile *Rochia nilotica*: Effects on Growth and Survival

**DOI:** 10.3390/ani16162537

**Published:** 2026-08-14

**Authors:** Jong-Seop Shin, Changju Lee, Yeong-Ji Park, Yu-Na Song, Han-Jun Kim, Sang-Woo Hur, June Kim, Hyun-Sung Yang

**Affiliations:** 1 Tropical & Subtropical Research Center, Korea Institute of Ocean Science & Technology (KIOST), Jeju 63349, Republic of Korea; 2 KIOST School, University of Science and Technology (UST), Daejeon 34113, Republic of Korea; 3 Research Cooperation Division, National Institute of Fisheries Science, Busan 46083, Republic of Korea; 4 Aquafeed Research Center, National Institute of Fisheries Science, Pohang 37517, Republic of Korea; 5 Korea·South Pacific Ocean Research Center, Korea Institute of Ocean Science & Technology (KIOST), Busan 49111, Republic of Korea

**Keywords:** marine resource, aquaculture, commercial top shell, *Sargassum horneri*, Chuuk, circular bioeconomy

## Abstract

*Rochia nilotica* is an economically important marine gastropod in Pacific Island countries, valued for its nacreous shell used in ornaments. The brown macroalga *Sargassum horneri* accumulates in large quantities along coastal regions, causing significant ecological and economic burdens. We evaluated the effects of *S. horneri*-based feed on the growth performance of juvenile *R. nilotica* under controlled tank conditions in Chuuk Lagoon, Micronesia. Juveniles (initial shell width ≈ 12.7 mm) were reared for 120 days and fed either a formulated diet (disc-shaped pellets, <1 cm in diameter) containing *S. horneri* or a traditional live-rock diet providing natural biofilms. Notable biological differences in shell width and height were observed after 2 months, with final mean values of 19.86 ± 1.76 mm and 15.92 ± 1.25 mm in the *S. horneri* group (94% survival), compared with 16.40 ± 1.26 mm and 13.14 ± 0.96 mm in the natural diet group (86% survival). Although greater growth and survival were observed in the tank receiving the formulated diet, fully replicated multi-tank experiments are required to definitively confirm whether these differences were caused directly by the dietary treatment. Given its abundance and environmental impact, recycling *S. horneri* biomass as a sustainable feed source could support trochid aquaculture and resource restoration and contribute to circular bioeconomy practices in Pacific Island countries.

## Simple Summary

The marine gastropod *Rochia nilotica* is a valuable resource for Pacific Island nations, but its wild populations are rapidly declining. Farming these snails using conventional tank-culture methods is challenging because their growth is inherently slow when relying solely on natural algae attached to tank surfaces. To address this, a formulated feed incorporating *Sargassum horneri*—a brown macroalga that causes harmful ‘Golden Tide’ blooms—was evaluated in a proof-of-concept investigation. Juvenile snails were fed either the natural algae or a diet containing 40% *S. horneri* for 120 days in a pilot-scale trial in Micronesia. The top shells fed the formulated diet grew notably faster in shell width and gained substantially more weight than those that consumed natural algae, with a high survival rate of 94%. These preliminary findings suggest that *S. horneri* can potentially be utilized in aquaculture feed to support sustainable production of *R. nilotica*, fostering a circular economy that benefits both the environment and local economies.

## 1. Introduction

*Rochia nilotica* (Tegulidae) is a large marine gastropod (shell width > 10 cm) widely distributed across Indo-Pacific coral reefs [1,2]. Besides its edible flesh, the species is highly valued for its thick nacreous shell, which is used to produce buttons and ornamental items [2]. Hence, *R. nilotica* has long been recognized as an important fishery and export commodity across Indo-Pacific region, including areas such as Micronesia, Indonesia, Australia, and Papua New Guinea [2,3,4,5,6]. However, due to overharvesting and habitat degradation, its global annual production has steadily declined from approximately 2300 t in the 1990s to 500 t in 2024 [7]. To mitigate this decline, countries such as Vanuatu, Indonesia, and the Philippines have implemented collection and export regulations alongside hatchery-based restocking programs [2]. Despite these efforts, the production of *R. nilotica* continues to rely on wild capture, underscoring the critical need for sustainable aquaculture approaches [7].

The artificial culture of *R. nilotica* generally follows a sequential process that comprises broodstock collection, induced or natural spawning, larval rearing, and juvenile grow-out [5,8]. In the wild, *R. nilotica* is herbivorous and feeds on benthic microalgae as well as small green and red macroalgae [9,10,11,12,13,14]. They show a distinct preference for soft filamentous algae and microalgae over tough, leathery brown algae [11]. Although this conventional method replicates wild foraging conditions by cultivating natural biofilms on artificial substrates for grazing, it is labor-intensive and yields relatively low productivity due to the inconsistent nutritional density of the biofilms. Given that juvenile *R. nilotica* exhibit inherently slow shell growth, typically 15–35 mm in shell width per year, and require 6–8 years to attain the commercial harvest size of 80–100 mm, the development of a nutritionally balanced formulated feed is essential to overcome this early grow-out bottleneck [9,13,15,16].

Finding sustainable and economically viable feed ingredients remains a major challenge in the development of formulated feeds. *Sargassum horneri* is a brown macroalga that blooms massively along the coasts of the Yellow Sea and East China Sea, causing recurrent “Golden Tide” events that severely affect marine ecosystems and the aquaculture industry [17,18,19]. In South Korea alone, an estimated 20,000–40,000 t of *S. horneri* drift ashore annually [17,20]. Although its massive accumulation is generally regarded as an environmental nuisance, the alga itself is non-toxic [21]. In fact, it is rich in minerals and bioactive compounds, including fucoidan, laminarin, and polyphenols, which are known to promote shell formation, antioxidant capacity, and immune responses in marine gastropods [22,23,24,25]. Upcycling this ecological residue into aquaculture feed offers a dual benefit by mitigating coastal pollution and reducing reliance on conventional feed ingredients. However, considering the natural aversion of *R*. *nilotica* to leathery brown macroalgae [10] and the digestive stress (e.g., reduced digestive enzyme activity) often caused by high *S*. *horneri* inclusion in other herbivores [26,27], its applicability for this species remains unclear.

Currently, commercially available formulated diets specifically designed for *R. nilotica* are lacking. Therefore, we aimed to evaluate the efficacy of upcycling *S. horneri* biomass into a formulated diet for juvenile *R. nilotica*. To evaluate the potential of *S. horneri* as a sustainable feed ingredient, the growth performance of juveniles fed a diet containing 40% *S. horneri* was compared with that of juveniles reared on traditional natural food sources under controlled pilot-scale conditions in Chuuk Lagoon, Micronesia. This approach is expected to improve hatchery productivity for resource restoration and support circular bioeconomy practices in Pacific Island regions.

## 2. Materials and Methods

### 2.1. Broodstock Collection and Larvae Preparation

Broodstock of *R. nilotica* was hand-collected via SCUBA diving from shallow coral reefs at approximately 5 m depth along the northeastern coast of Weno Island, Chuuk State, Federated States of Micronesia (7.46° N, 151.90° E) in April 2024. The rearing experiment was conducted at the Korea·South Pacific Ocean Research Center (KSORC), Weno, Chuuk. Spawning was induced following the protocol of Nash [5]. After collection, the broodstock shells were cleaned, and the animals were placed in a flow-through tank. To simulate the natural nocturnal spawning behavior of this species, the tank was kept in complete darkness for 40 h adapting the protocol described by Nash [5]. Once spawning had occurred in both males and females, the inflow pump was stopped to allow fertilization to occur within the tank. Successful fertilization was subsequently confirmed by observing early cell cleavage in egg samples under a light microscope.

### 2.2. Experimental Diets

The *S. horneri* biomass used in this study was collected from the coastal areas of Jeju Island, Korea, during the ‘Golden Tide’ bloom in Spring 2024. Immediately after collection, the algal biomass was thoroughly washed multiple times with fresh water to remove sand, attached epiphytes, and excess surface salts. The cleaned biomass was then dried using a far-infrared dryer until a constant weight was achieved, and subsequently ground into a fine powder using a laboratory mill. The experimental diet was formulated to contain 40% *S. horneri* powder, with 11% fish meal, 20% corn gluten meal, and 2.3% defatted soybean meal as the main protein sources. Dextrin (1.6%) and wheat flour (20%) were used as carbohydrate sources, and fish oil (1.7%) was incorporated as the lipid source [28]. All ingredients were thoroughly mixed, pelleted using a laboratory pellet extruder. The resulting feed was formed into extruded, smooth round-edge flakes with a surface area of approximately 10 mm^2^. These flakes were dried in an electric drying oven for 3 h, subsequently sealed in polyethylene bags, and stored at −20 °C until use. The proximate composition of the formulated diet (dry matter basis) was 33.4% crude protein, 3.98% crude lipid, and 18.63% ash (Table 1).

### 2.3. Feeding Trial

To evaluate the effects of the experimental diet, juvenile *R. nilotica* (mean shell width ≈ 12.7 mm; mean body weight ≈ 0.46 g) were divided into two dietary treatments: a formulated diet group (*S. horneri*) and a natural food sources group (NFS). Due to space and facility limitations at the Chuuk field station, this experiment was conducted as a pilot study using a single 200-L tank per treatment. Juveniles were stocked at 50 individuals per tank. Any dead individuals were removed daily and not replaced; therefore, the stocking density slightly decreased over the experimental period in accordance with the final survival rates (94% in the formulated diet group and 86% in the NFS group).

For the *S. horneri* group, a new flow-through tank without live rocks or attached algae was prepared. For the NFS group, live rocks (coral fragments) naturally colonized by benthic microalgae and periphyton from natural seawater were supplied to the tank. Prior to introduction, these rocks were visually inspected and gently rinsed with filtered seawater to remove any potential predators or large unwanted macrobenthos while preserving the biofilm. The surface area and volume of the live rocks were visually monitored daily to be sufficient to maintain a continuous natural food supply throughout the trial. Although microalgal biomass was not quantitatively measured, this continuous monitoring helped to minimize the likelihood that growth differences would reflect absolute food deprivation rather than the nutritional density of the diets.

The feeding trial was conducted for 120 days, from 23 August to 18 December 2024. For the formulated diet group, feed was provided once daily at 18:00 at a ration equivalent to 1.5% of the total biomass per tank. This feeding rate was adopted based on established protocols for related marine herbivorous gastropods fed artificial diets [29]. Uneaten feed and feces were removed at 08:00 the following morning strictly to maintain water quality, and were not quantified for daily feed consumption or feed conversion ratio calculations. Throughout the feeding trial, the rearing water parameters were strictly monitored using a multiparameter water quality meter (ProDSS, YSI Inc., Yellow Springs, OH, USA). The conditions were maintained at a temperature of 29.0 °C, dissolved oxygen of 6.2 ± 0.3 mg/L, salinity of 33.3 ± 0.4 psu, and pH of 8.2 ± 0.2.

### 2.4. Growth Measurement

Biomass was measured monthly by removing all *R. nilotica* individuals from each tank. Shell width (SW; the maximum width of the shell measured perpendicular to the shell axis) and shell height (SH; the vertical distance from the apex of the protoconch to the shell base) were measured using a Vernier caliper with a precision of 0.01 mm. Total weight (TW) was determined by weighing all individuals in each tank collectively and dividing the total weight by the number of surviving individuals to obtain the mean weight per individual. This collective weighing approach was necessary due to the lack of high-precision balances capable of rapidly weighing individual small juveniles at the field station. Consequently, formal statistical analysis was not performed for TW.

Growth performance parameters, including shell width growth rate (SWGR), daily increment in shell width (DISW), shell height growth rate (SHGR), daily increment in shell height (DISH), weight growth rate (WGR), specific growth rate in weight (SGRW), and survival rate (SR), were calculated as follows:

SWGR (%) = [(FSW − ISW)/ISW] × 100
DISW (μm/day) = [(FSW − ISW)/days] × 1000
SHGR (%) = [(FSH − ISH)/ISH] × 100
DISH (μm/day) = [(FSH − ISH)/days] × 1000
WGR (%) = [(FW − IW)/IW] × 100
SGRW (%/day) = [(ln(FW) − ln(IW))/days] × 100
SR (%) = [(final number of *R. nilotica*)/(initial number of *R. nilotica*)] × 100

where FSW and ISW are the final and initial shell widths, FSH and ISH are the final and initial shell heights, FW and IW are the final and initial TW, respectively, and ‘days’ is the total rearing period (120 days).

### 2.5. Data Analysis and Presentation

Due to logistical constraints at the field station, this pilot study was conducted using a single tank per dietary treatment. We explicitly acknowledge that applying dietary treatments to single tanks and treating individual *R. nilotica* (*n* = 43–50) as independent biological replicates constitutes pseudoreplication [30]. Consequently, potential tank effects cannot be completely decoupled from dietary treatment effects in this experimental design. Therefore, no formal statistical hypothesis testing (e.g., *t*-tests) or *p*-value calculations were performed to compare the two groups. Instead, all data are presented strictly in an exploratory and descriptive manner to illustrate the observed biological trends within this specific trial. Growth parameters (shell width and shell height) are reported as the mean ± standard deviation (S.D.) of the surviving individuals in each tank.

## 3. Results

### 3.1. Monthly Changes in Growth

**Figure 1:**
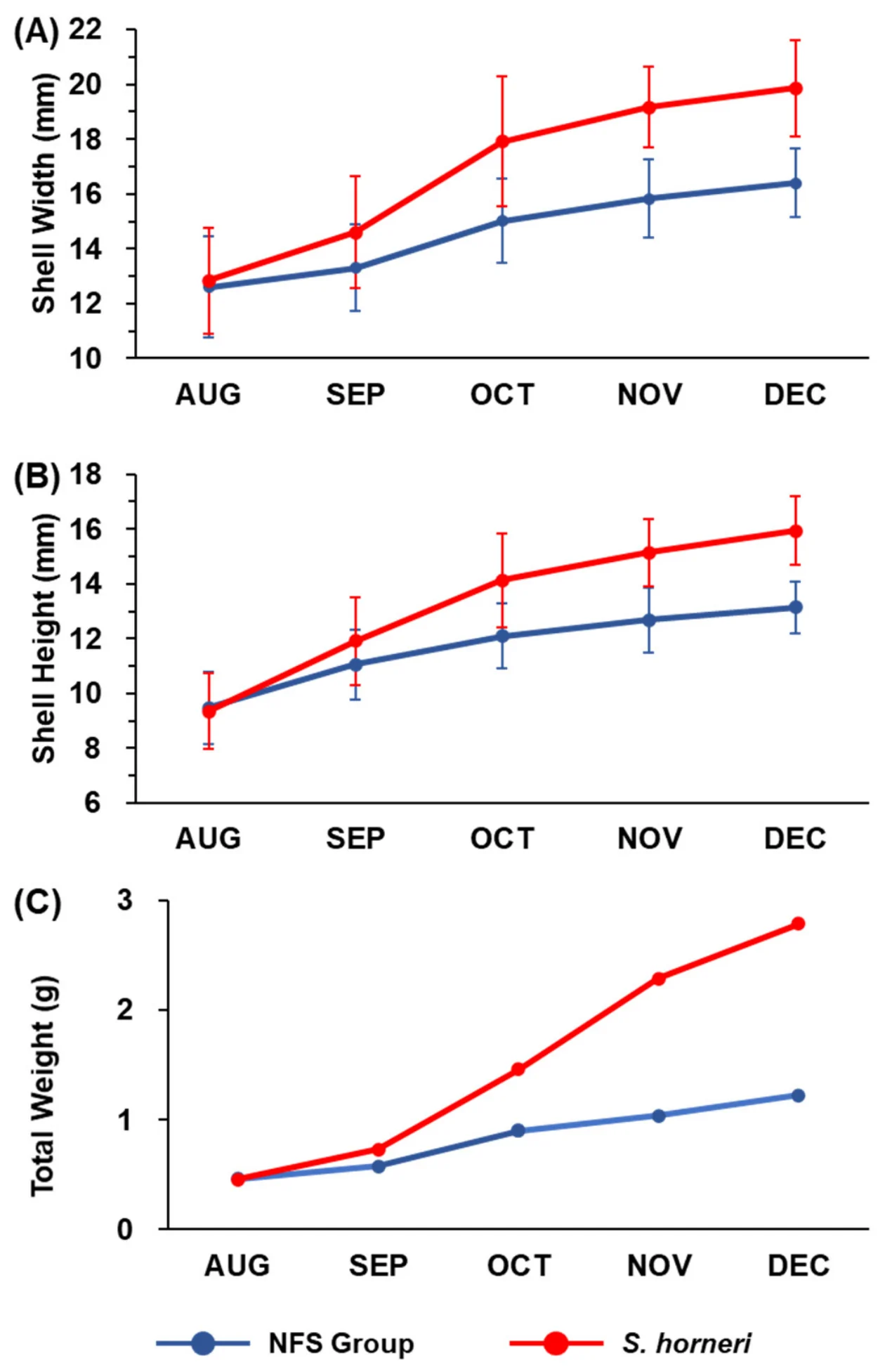
Monthly growth performance of *R. nilotica* juveniles under two feeding regimes from August to December 2024 (120 days). (**A**) Shell Width (mm), (**B**) Shell Height (mm), and (**C**) Total Weight (g). Values are monthly means ± S.D. (*n* varied between 43 and 50 due to mortality).

During the 120-day exploratory feeding trial, mean SW and SH were notably higher in the *S. horneri*-fed group compared with the NFS group. While the mean sizes between the two tank cohorts were similar during the first month of feeding, a noticeable divergence in growth occurred from the second month onward, with the *S. horneri*-fed group consistently exhibiting larger mean SW and SH (Figure 1A,B).

Regarding biomass, juveniles fed *S. horneri* exhibited a notable numerical increase in mean TW compared to the NFS group after two months of feeding. The estimated mean TW of the *S. horneri*-fed group exceeded that of the NFS by approximately 162.2%, 220.2%, and 228.7% after 2, 3, and 4 months of culture, respectively (Figure 1C).

### 3.2. Effect of S. horneri-Based Diet on Growth Performance

Table 2 summarizes the effects of feeding trial on the growth of *R. nilotica*. After the 4-month period, all growth parameters increased markedly in the *S. horneri* treatment compared with those of the NFS group. At the end of the experiment, the final shell width (FSW) reached 16.40 ± 1.26 mm in the NFS group and 19.86 ± 1.76 mm in the *S. horneri* group. The shell width growth rate (SWGR) was 30.06% (31.6 µm/day) for the NFS group and 54.79% (58.6 µm/day) for the *S. horneri* group, indicating approximately 1.8-fold enhancement.

Final shell height (FSH) increased to 13.14 ± 0.96 mm in the NFS group and 15.92 ± 1.25 mm in the *S. horneri* group. The corresponding SHGR was 38.61% and 70.27%, respectively. Daily increment in shell height (DISH) was likewise greater in the *S. horneri* group (54.8 µm/day) than in the NFS group (30.5 µm/day).

The most pronounced biological effect size was in body weight. Final mean weight (FW) increased from 1.22 g in the NFS group to 2.79 g in the *S. horneri* group. Hence, WGR and SGRW were 165.22% (0.81%/day) and 506.52% (1.51%/day) for the two groups, respectively, indicating a substantial improvement in growth efficiency under *S. horneri* feeding conditions. The survival rates in both treatments were high throughout the experimental period, with survival rates of 86% in the NFS group and 94% in the *S. horneri* group.

## 4. Discussion

The present preliminary study provides initial evidence suggesting that upcycling *S. horneri* ecological residues into a formulated diet may enhance early growth in juvenile *R. nilotica*. Compared with juveniles on natural food sources (NFS), the group fed the 40% *S. horneri* diet exhibited an approximately 1.8-fold higher shell width (SW) and a substantial increase in total biomass (Figure 1). Although a relatively high survival rate (86%) was maintained in the NFS group (Table 1), the higher survival (94%) observed in the *S. horneri*-fed group suggests that traditional natural food sources may still present subtle nutritional limitations. This indicates that the enhanced nutritional profile of the formulated diet is highly beneficial not only for driving early growth but also for maximizing overall survival in tank-culture systems.

Previous feeding preference studies have demonstrated that *R. nilotica* naturally consumes both microalgae and macroalgae [10,12,15] but exhibits strong preferences based on the structural characteristics (e.g., structural toughness and thallus morphology) and nutritional profiles (e.g., nutrient value and chemical defenses) of the diets [10]. Benthic microalgae, such as diatoms, generally contain higher percentages of protein and lipid and lower levels of inorganic ash compared to macroalgae [11,31,32,33]. Thus, microalgae are highly preferred and easily grazed by *R. nilotica*. Conversely, natural macroalgae provide necessary carbohydrates, but tough brown algae are usually the least preferred food source due to their structural grazing difficulty and high inorganic matter content [10,11,21]. The results of the present study highlight the advantage of the formulated diet. By mechanically processing the leathery brown alga *S. horneri* into a powder and formulating it with high-quality protein and lipid sources such as fish meal and fish oil, the structural barriers of the macroalga were substantially reduced. This formulated approach allowed the juveniles to capitalize on the abundant minerals and bioactive compounds of *S. horneri* while simultaneously receiving the high protein and lipid densities typically associated only with preferred microalgal diets.

Nutritionally, *S. horneri* contains free amino acids such as serine and aspartic acid, which act as natural feeding stimulants of mollusk chemoreceptors [34], to promote active feed intake. Alongside these, bioactive substances like fucoidan and phlorotannins offer robust antioxidant and anti-inflammatory benefits [35,36,37,38,39] which are highly relevant for mitigating physiological stress, enhancing immune responses, and supporting the overall health and survival of juvenile gastropods in tank-culture systems. Notably, recent studies on abalone (*Haliotis discus hannai*) revealed that the replacement of conventional macroalgae with *S. horneri* at levels above 50% leads to severe growth retardation and pathological damage to the digestive gland, such as abnormal vacuole expansion and nuclear lysis, due to the limited digestibility of non-starch polysaccharides and phenolic compounds [24,34]. However, *R. nilotica* juveniles in this study tolerated a 40% inclusion level without any observable detrimental effects on growth or survival. This suggests that *R. nilotica*, an active grazer with a distinct evolutionary feeding ecology, may possess a higher physiological tolerance for complex macroalgal components than abalone [10,27], possibly reflecting its highly active foraging behavior and the diverse range of algal species it naturally consumes on coral reefs.

Furthermore, the efficient digestion of such high levels of macroalgal biomass is likely facilitated by the intestinal microbiome [24,29,40]. In various other marine herbivorous gastropods (e.g., abalone), bacterial taxa such as *Psychrilyobacter* and *Mycoplasma* have been widely documented to assist in breaking down complex carbohydrates and enhancing nutrient assimilation [29,40]. Although their specific roles in *R. nilotica* remain to be fully characterized, similar microbiome-mediated digestive mechanisms likely contribute to their tolerance of macroalgal diets [41,42]. Therefore, the observed growth improvement may be attributed not only to the basal nutritional value of the diet but also to microbiome-mediated enhancements in energy metabolism. To validate this hypothesis, future research on 16S rRNA amplicon sequencing is necessary to directly characterize the gut microbial shifts induced by *S. horneri* consumption in *R. nilotica*.

It is crucial to acknowledge the logistical constraints and the exploratory nature of the present pilot study. Due to facility limitations at the Chuuk field station, the experiment was conducted using a single tank per dietary treatment. As a result, individual snails were treated as observational units, which introduces pseudoreplication. Because of this design, the observed growth enhancements cannot be definitively isolated from potential tank-specific environmental effects (i.e., tank effects). Therefore, the statistical comparisons and conclusions drawn herein should be interpreted as exploratory indicators of biological effect sizes rather than confirmatory evidence of causal dietary effects. Future studies employing fully replicated multi-tank designs are required to statistically validate weight gain metrics and definitively confirm the efficacy of the formulated diet.

Nevertheless, the successful incorporation of 40% *S. horneri* into *R. nilotica* feed presents a compelling paradigm for circular bioeconomy practices in aquaculture. The massive drift and accumulation of *S. horneri*, known as “Golden Tides,” causes recurrent ecological and economic damage and requires substantial administrative budgets for biomass collection and disposal [43,44]. Upcycling this environmental nuisance into a functional feed ingredient offers a dual-purpose sustainable strategy: it drastically mitigates coastal residue management costs and provides a locally sourced, renewable nutritional resource that substitutes costly imported feed ingredients. Moreover, to safely push the dietary inclusion rates beyond 40% in the future, advanced pre-processing techniques, such as hot-water extraction and *Lactiplantibacillus pentosus*-mediated fermentation, should be investigated to break down rigid cell walls and neutralize the bioaccumulation risk of inorganic arsenic inherent in *Sargassum* species [45,46]. Furthermore, given the current lack of species-specific formulated feeds, future studies evaluating specific digestive enzyme activities are highly recommended to better understand the digestive physiology of *R. nilotica* and facilitate the development of optimized diets. Overall, this advanced upcycling approach can improve hatchery productivity, fortify the economic resilience of the aquaculture sector, and support sustainable stock enhancement programs that are vital for coastal livelihoods and local economies in Pacific Island nations, ultimately aligning with broader carbon-neutral ESG goals.

## 5. Conclusions

This pilot study suggests that upcycling the brown macroalga *S. horneri* into a formulated diet (40% inclusion) could be a viable strategy for the early grow-out of juvenile *R. nilotica*. Noticeably greater shell width, shell height, and biomass accumulation were observed in the tank receiving the *S. horneri*-formulated diet compared with the tank relying on traditional natural food sources, suggesting a potential to effectively overcome the nutritional bottlenecks of conventional tank-culture methods. Furthermore, the successful utilization of *S. horneri* ecological residues offers a sustainable, dual-purpose solution of mitigating the ecological and economic impacts of “Golden Tide” macroalgal blooms and providing a renewable, locally sourced feed ingredient for trochid aquaculture. Although constrained by a single-tank experimental design, these preliminary findings strongly support the integration of nuisance macroalgal biomass into circular bioeconomy frameworks. As noted, fully replicated multi-tank experiments are required to definitively confirm whether the observed growth differences were caused directly by the dietary treatment. Future research should prioritize these replicated trials and investigate the microbiome-mediated digestive mechanisms underlying this enhanced growth performance.

## Figures and Tables

**Table 1:** Ingredient and chemical composition of the experimental diets (%, DM basis).

Ingredients	Contents (%)
Fish Meal	11.00
*S. horneri*	40.00
Corn Gluten Meal	20.00
Defatted Soybean Meal	2.30
Wheat Flour	20.00
Dextrin	1.60
Vitamin Mix *	0.90
Mineral Mix **	2.00
Choline	0.50
Fish Oil	1.70
Proximate Analysis (% Dry Matter)	
Crude Protein	33.4
Crude Lipid	3.98
Ash	18.63

* Vitamin mixture composition (unit kg^−1^): ascorbic acid, 6400 mg; tocopherol acetate, 37,500 mg; thiamin nitrate, 5000 mg; riboflavin, 10,000 mg; pyridoxine hydrochloride, 5000 mg; nicotinic acid, 37,500 mg; Ca-D-pantothenate, 17,500 mg; inositol, 75,000 mg; biotin, 50 mg; folic acid, 2500 mg; menadione sodium bisulfite, 2500 mg; retinol acetate, 5,000,000 IU; cholecalciferol, 1,000,000 IU; cyanocobalamin, 25 mg. ** Mineral mixture composition (g kg^−1^): ferrous fumarate, 12.5; manganese sulfate, 11.3; ferrous sulfate, 20.0; cupric sulfate, 1.25; cobaltous sulfate, 0.75; zinc sulfate, 13.75; calcium iodate, 0.75; magnesium sulfate, 80.2; aluminum hydroxide, 0.75.

**Table 2:** Dietary effect on the growth performance of *R. nilotica* juveniles after 4 months of feeding trials with *S. horneri*-based diet and NFS group. Values are expressed as mean ± S.D.

	NFS Group	*S. horneri*
ISW (mm)	12.61 ± 1.82	12.83 ± 1.94
FSW (mm)	16.40 ± 1.26	19.86 ± 1.76
SWGR (%)	30.06	54.79
DISW (µm/day)	31.58	58.58
ISH (mm)	9.48 ± 1.32	9.35 ± 1.38
FSH (mm)	13.14 ± 0.96	15.92 ± 1.25
SHGR (%)	38.61	70.27
DISH (µm/day)	30.50	54.75
IW (g)	0.46	0.46
FW (g)	1.22	2.79
WGR (%)	165.22	506.52
SGRW (%/day)	0.81	1.51
SR (%)	86	94

ISW and FSW: initial and final shell width; ISH and FSH: initial and final shell height; IW and FW: initial and final body weight; SWGR, SHGR, and WGR: shell width, shell height, and weight growth rates; DISW and DISH: daily increment in shell width and daily increment in shell height; SGRW: specific growth rate in weight; SR: survival rate.

## Data Availability

The original raw data supporting the conclusions of this article, including individual shell measurements, monthly survival, and tank biomass, will be made available by the corresponding author upon reasonable request.
